# Association of Ocular Surface Diseases With SARS-CoV-2 Infection in Six Districts of China: An Observational Cohort Study

**DOI:** 10.3389/fimmu.2021.695428

**Published:** 2021-08-06

**Authors:** Shengjie Li, Yichao Qiu, Li Tang, Zhujian Wang, Wenjun Cao, Xingtao Zhou, Xinghuai Sun

**Affiliations:** ^1^Clinical Laboratory, Eye & ENT Hospital, Fudan University, Shanghai, China; ^2^Eye Institute and Department of Ophthalmology, Eye & ENT Hospital, Fudan University, Shanghai, China; ^3^NHC Key Laboratory of Myopia (Fudan University), Key Laboratory of Myopia, Chinese Academy of Medical Sciences, Shanghai, China; ^4^Shanghai Key Laboratory of Visual Impairment and Restoration, Shanghai, China; ^5^Shanghai Research Center of Ophthalmology and Optometry, Shanghai, China; ^6^Shanghai Engineering Research Center of Laser and Autostereoscopic 3D for Vision Care (20DZ2255000), Shanghai, China

**Keywords:** SARS- CoV-2, immunoglobulin G, immunoglobulin M, ocular surface diseases, cross-sectional study, seroprevalence, COVID - 19

## Abstract

The severe acute respiratory syndrome coronavirus 2 (SARS-CoV-2) viruses is mainly transmitted through respiratory droplets. Notably, some coronavirus disease 2019 (COVID-19) patients have ocular manifestations, including conjunctival hyperaemia, chemosis, epiphora, and increased secretions. However, the association between SARS-CoV-2 and ocular surface diseases is poorly described. Between May 2020 and March 2021, a total of 2, 0157 participants from six districts of China were enrolled. Serum samples were tested for immunoglobulin G and M (IgG and IgM) antibodies against the SARS-CoV-2 spike protein and nucleoprotein using magnetic chemiluminescence enzyme immunoassays. Throat swabs were tested for SARS-CoV-2 RNA using RT-PCR assays in a designated virology laboratory. Fisher exact, χ^2^ test, and logistic regression analysis were performed. Of 2, 0157 serum samples tested, 1, 755 (8.71%) were from ocular surface diseases, 1, 2550 (62.26%) from no-ocular surface diseases (ocular diseases except ocular surface diseases), 5, 852 (29.03%) from no-ocular diseases. SARS-CoV-2 prevalence for the combined measure was 0.90% (182/2, 0157). Seroprevalence of SARS-CoV-2 was significantly (p<0.05) higher in the population with ocular surface diseases (2.28%, 40/1755) compared with no-ocular surface diseases (0.70%, 88/1, 2550), and no-ocular diseases (0.92%, 54/5, 852). Similar results were also observed with respect to sex, age, time, and districts. Logistic regression analyses revealed that ocular surface diseases [ocular surface diseases *vs.* no-ocular diseases (p=0.001, OR =1.467, 95% CI=1.174-1.834); ocular surface diseases *vs.* no-ocular surface diseases (p<0.001, OR =2.170, 95% CI=1.434-3.284)] were associated with increased risk of susceptible to SARS-CoV-2 infection. In a word, there was a significant association between ocular surface disease and SARS-CoV-2 infection. Therefore, increasing awareness of eye protection during the pandemic is necessary, especially for individuals with ocular surface diseases.

## Introduction

Coronavirus disease 2019 (COVID-19), a novel respiratory disease caused by severe acute respiratory syndrome coronavirus 2 (SARS-CoV-2), emerged in December 2019. As of July 13, 2021, 187,086,096 laboratory-confirmed cases of COVID-19 were reported, with more than 4,042,921 known fatalities (https://covid19.who.int/). The SARS-CoV-2 virus is mainly transmitted through respiratory droplets and by close contact with infected individuals (1). Notably, some patients have ocular manifestations, including conjunctival hyperaemia, chemosis, epiphora, and increased secretions (2–4). Furthermore, a recent study reported that the SARS-CoV-2 receptor angiotensin-converting enzyme 2 (ACE2) and entry protease transmembrane serine protease 2 (TMPRSS2) are strongly detected in human ocular surfaces, indicating that they may provide SARS-CoV-2 with a portal of entry (5). Thus, ocular surface may as a possible site of virus entry and also as a source of contagious infection.

Little information is available about the prevalence of SARS-CoV-2 in population with ocular diseases, and the association between SARS-CoV-2 and ocular surface diseases is poorly described. To our knowledge, only one study performed a retrospective small sample (n=1100) survey found no association between ocular symptoms and COVID-19 positivity in an outpatient population (6). Therefore, estimating the seroprevalence of SARS-CoV-2 in patients with ocular surface diseases is crucial for controlling the transmission of SARS-CoV-2 through ocular surface.

Serological tests, such as validated assays for immunoglobulin G (IgG) and immunoglobulin M (IgM) antibodies against SARS-CoV-2, have the advantages of easy serum sample collection and high throughput (7). Antibodies can be detected in most COVID-19 patients 7-14 days after diagnosis (8, 9) and remain at high levels four months after diagnosis (10). In this study, we assayed IgG and IgM antibodies to estimate the seropositivity rates to assess the association of ocular surface diseases with SARS-CoV-2 infection.

## Methods

### Study Design and Participants

**Figure 1:**
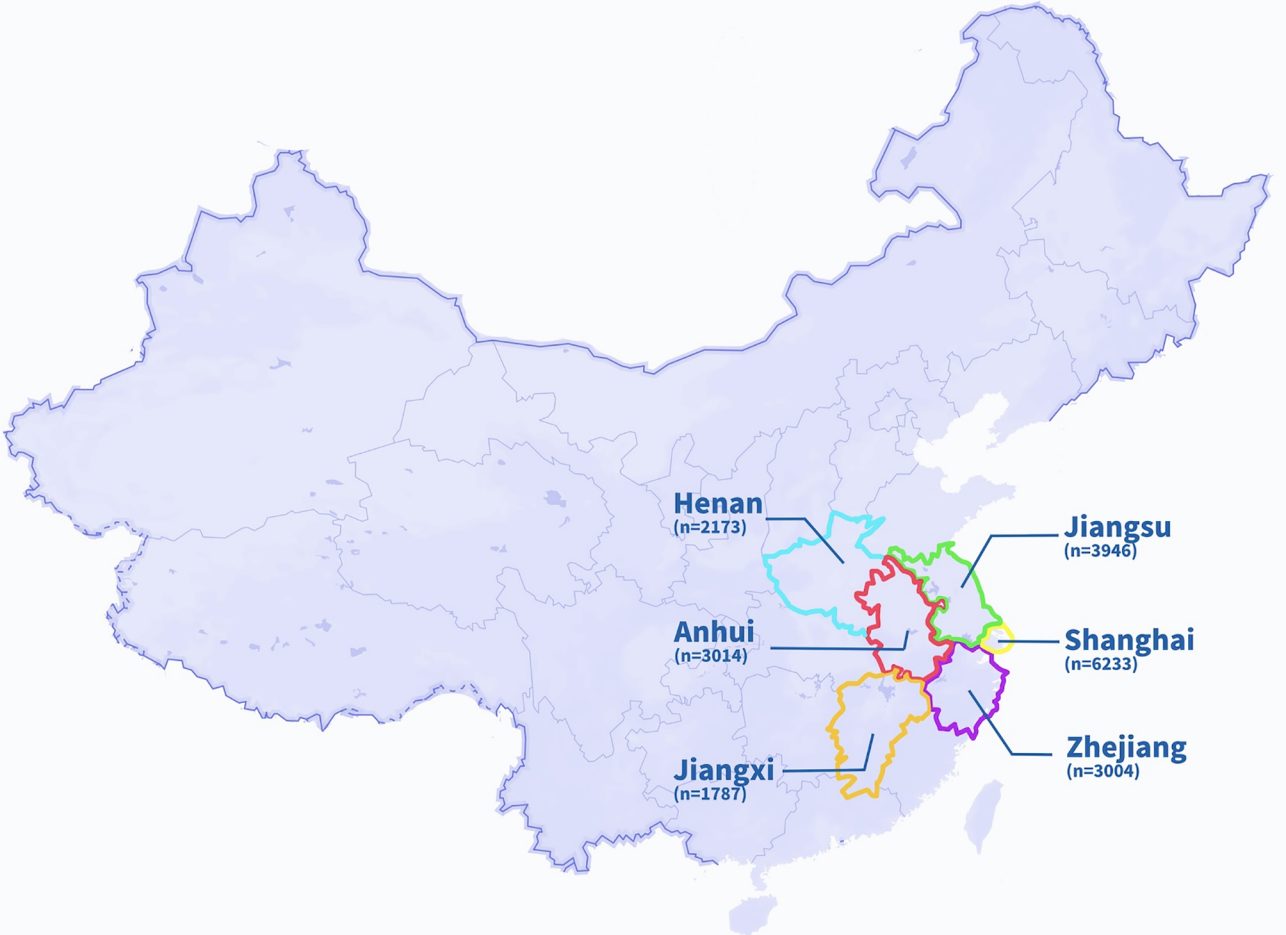
Locations of the six districts in China.

A total of 2,0157 individuals were enrolled in this study from May 2020 to March 2021: 6,233 from Shanghai, 3,946 from Jiangsu province, 3,014 from Anhui province, 3,004 from Zhejiang province, 1,787 from Jiangxi province, and 2,173 from Henan province (**Figure 1** and **Table 1**). The subjects who have been vaccinated against SARS-CoV-2 were excluded from this study (n=81).

**Table 1:** Number of clinical specimens for antibodies to SARS-CoV-2 in six districts.

	Shanghai	Jiangsu	Anhui	Henan	Jiangxi	Zhejiang
Sex						
Male	3021	1961	1538	1012	800	1361
Female	3212	1985	1476	1161	987	1643
Age, y						
0-18	356	279	219	123	85	236
19-49	2227	1913	1283	685	719	1361
50-64	1722	994	832	646	547	794
≥65	1928	760	680	719	436	613
Total	6233	3946	3014	2173	1787	3004

All subjects underwent medical examinations performed by respective specialty physicians. Meanwhile, all subjects underwent ophthalmic examination conducted by an ophthalmologist specialist. The ocular diseases were diagnosed by an ophthalmologist. The time frame of the ocular condition was also recorded. In the present study, the patients with ocular diseases were newly diagnosed between May 2020 to March 2021 when they came to the hospital, and the blood of the patients was collected and measured immediately.

The ocular surface diseases group included corneal diseases, conjunctival diseases, dry eye, eyelid disease, lacrimal apparatus diseases, *etc.* The no-ocular surface diseases group included ocular diseases except ocular surface diseases. The no-ocular diseases group included all the subjects except patients with ocular diseases.

### Ethics Approval

The Medical Ethics Committees of Eye and ENT Hospital of Fudan University approved this study, and the study adhered to the principles of the Declaration of Helsinki. Informed consent was obtained from all participants. For individuals younger than 18 years, consent was provided by parents or a legal guardian.

### Laboratory Measurements

Throat swab samples were collected from May 2020 through March 2021. All samples were immediately transported to the laboratory to maintain the cold chain and tested within 12 hours of collection. Throat swabs were tested for SARS-CoV-2 RNA using RT-PCR assays in a designated virology laboratory (Zhongke Runda, Shanghai, China).

Serum samples were also collected from May 2020 through March 2021. All samples were analysed within eight hours of collection. IgG and IgM antibodies against the SARS-CoV-2 spike protein and nucleoprotein were measured using a commercially available magnetic chemiluminescence enzyme immunoassay kit (Bioscience, Chongqing, China), per the manufacturer’s instructions. Antibody levels were expressed as the ratio of the chemiluminescence signal to the cut-off (S/CO) value. An S/CO value higher than 1 for either IgG or IgM was considered positive. If the S/CO value was higher than 0.7 but lower than 1.2, the serum sample was re-analysed to confirm the results. Serological assays for SARS-CoV-2 infection have been validated in a previous study (11), which as follow: serum of 447 patients with end-stage kidney disease that were collected as a negative control and serum of 242 patients with COVID-19 confirmed by the viral RT–PCR test as a positive control and calculated the sensitivity and specificity of the assay. The serological test showed specificity of 99.3% (444 of 447) and 100% (447 of 447) for IgG and IgM antibodies, respectively. Moreover, interference test reported that rheumatoid factor (1500 IU/ml), non-specific IgG antibody (38.8g/l), on-specific IgM antibody (4.8g/l), antinuclear antibodies, and anti-mitochondrial antibody didn’t have the potential interference on the results.

Since the IgG and IgM levels should decrease as time passed from the infection. Thus, we set the S/CO value at 0.5 levels for either IgG or IgM which might be infected with COVID-19 in the past (**Table S1**).

### Statistical Analysis

A sample size calculation was undertaken in order to determine the study’s recruitment sample sizes. We used an open-source calculator to calculate the minimal required sample size based on the probability of a type I error of alpha at 5% (two-sided), type II error of beta at 30%, and the normal prevalence of COVID-19 among the population in China, which is approximately 1%. This calculation yielded a sample consisting of at least 5,293 participants. Categorical variables were presented as counts and percentages. Fisher exact and χ^2^ test was used for comparison between the groups. The results of logistic regression were presented as OR and corresponding 95% confidence intervals (CIs). All analyses were performed using the Statistical Package for the Social Sciences software, version 13.0 (SPSS Inc., Chicago, IL, USA). Figures were created using GraphPad Prism 6 (GraphPad Software, La Jolla, CA, USA). A value of P < 0.05 was considered statistically significant.

## Results

**Figure 2:**
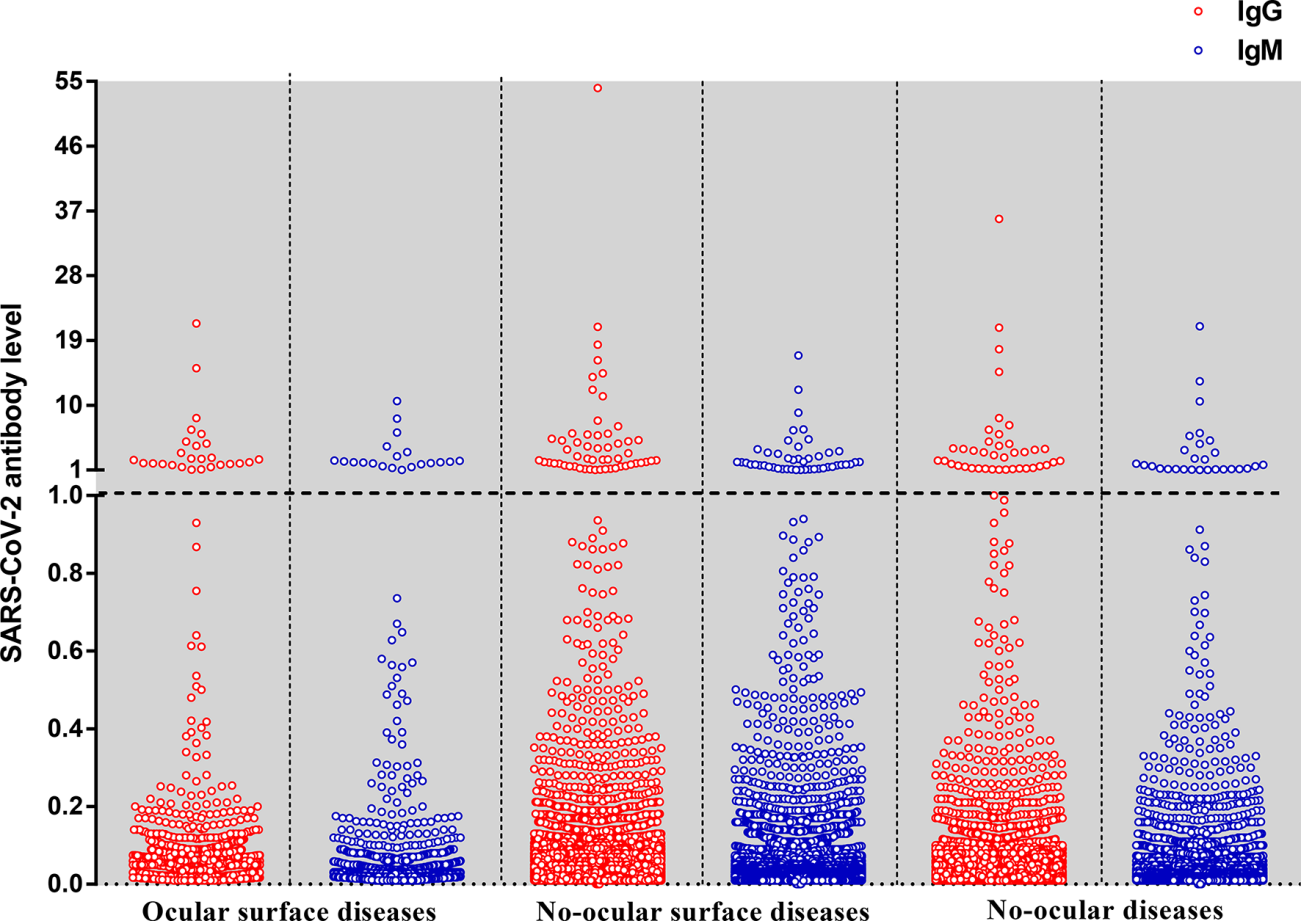
The level of IgG and IgM antibodies against the nucleoprotein and a peptide from the spike protein of SARS-CoV-2 by the type of diseases. Each dot represents the level of IgG (red) or IgM (blue) antibody for an individual. The SARS-CoV-2 antibody level expressed as the ratio is calculated by the chemiluminescence signal divides the cutoff value (S/CO). The dotted line means that the antibody level is at the cutoff value (S/CO=1).

Of all specimens, 1, 755 (8.71%) were from ocular surface diseases, 1, 2550 (62.26%) from no-ocular surface diseases, 5, 852 (29.03%) from no-ocular diseases (**Table 2**). In this study, all the participants were RT-PCR negative for SARS-CoV-2 from throat swabs. The levels of IgM and IgG antibodies are presented in **Figure 2**. SARS-CoV-2 prevalence for the combined measure was 0.90% (182/2, 0157). The seroprevalence in ocular surface diseases, no-ocular surface diseases (ocular diseases except ocular surface diseases), and no-ocular diseases was 2.28% (40/1755), 0.70% (88/12550), and 0.92% (54/5852), respectively. The seroprevalence of antibodies to SARS-CoV-2 was particularly high in the population with ocular surface diseases (p<0.05) (**Table 2**). Furthermore, the seroprevalence of IgM ^+^ (1.42%, 0.39%, 0.53%) and IgG ^+^ (1.25%, 0.43%, 0.68%) was also particularly high in the population with ocular surface diseases (p<0.05) compared with no-ocular surface diseases and no-ocular diseases, respectively (**Table 2**). Owing to the ocular surface diseases are composed with various diseases, we further analysis the seroprevalence of antibodies to SARS-CoV-2 among different types of ocular surface diseases (**Table 3**).

**Table 2:** SARS-CoV-2 seroprevalence by sex and districts.

	n	IgG^+^	IgM^+^	IgM^+^ or IgG^+^
Ocular surface diseases	1755	22 (1.25%)	25 (1.42%)	40 (2.28%)
Gender				
Male	699	9 (1.29%)	10 (1.43%)	14 (2.00%)
Female	1056	13 (1.23%)	15 (1.42%)	26 (2.46%)
Districts				
Shanghai	591	8 (1.35%)	5 (0.84%)	11 (1.86%)
Jiangsu	386	3 (0.78%)	7 (1.81%)	9 (2.33%)
Anhui	96	2 (2.08%)	1 (1.04%)	3 (3.13%)
Henan	242	2 (0.83%)	4 (1.65%)	5 (2.07%)
Jiangxi	136	2 (1.47%)	1 (0.74%)	3 (2.21%)
Zhejiang	304	5 (1.64%)	7 (2.30%)	9 (2.96%)
No-ocular surface diseases	12550	54 (0.43%)	49 (0.39%)	88 (0.70%)
Gender				
Male	6242	20 (0.32%)	21 (0.34%)	34 (0.54%)
Female	6308	34 (0.54%)	28 (0.44%)	54 (0.86%)
Districts				
Shanghai	4607	13 (0.28%)	16 (0.35%)	25 (0.54%)
Jiangsu	2321	9 (0.39%)	7 (0.30%)	15 (0. 65%)
Anhui	2089	8 (0.38%)	8 (0.38%)	14 (0.67%)
Henan	1322	7 (0.53%)	6 (0.45%)	11 (0.83%)
Jiangxi	966	5 (0.52%)	4 (0.41%)	8 (0.83%)
Zhejiang	1245	12 (0.96%)	8 (0.64%)	15 (1.20%)
No-ocular diseases	5852	40 (0.68%)	31 (0.53%)	54 (0.92%)
Gender				
Male	2752	16 (0.58%)	15 (0.55)	20 (0.73%)
Female	3100	24 (0.77%)	16 (0.52%)	34 (1.10%)
Districts				
Shanghai	1035	7 (0.68%)	7 (0.68%)	9 (0.87%)
Jiangsu	1239	6 (0.48%)	6 (0.48%)	10 (0.81%)
Anhui	829	7 (0.84%)	2 (0.24%)	7 (0.84%)
Henan	609	5 (0.82%)	3 (0.49%)	5 (0.82%)
Jiangxi	685	7 (1.02%)	3 (0.44%)	7 (1.02%)
Zhejiang	1455	8 (0.55%)	10 (0.69%)	16 (1.10%)
Total	20157	116 (0.58%)	105 (0.52%)	182(0.90%)

**Table 3:** SARS-CoV-2 seroprevalence among different type of ocular surface diseases.

	n	IgG^+^	IgM^+^	IgM^+^ or IgG^+^
Ocular surface diseases	1755	22 (1.25%)	25 (1.43%)	40 (2.28%)
Xerophthalmia	174	4 (2.30%)	4 (2.30%)	6 (3.45%)
Keratitis	231	3 (1.30%)	3 (1.30%)	4 (1.73%)
Conjunctival cyst	80	0 (0%)	3 (3.75%)	3 (3.75%)
Conjunctivitis	87	3 (3.45%)	3 (3.45%)	5 (5.75%)
Dacryocystitis	117	1 (0.85%)	2 (1.71%)	3 (2.56%)
Conjunctival congestion	97	0 (0%)	2 (2.06%)	2 (2.06%)
Trichiasis	105	2 (1.90%)	0 (0%)	2 (1.90%)
Pterygium	201	6 (2.99%)	4 (1.99%)	8 (3.98%)
Conjunctival nevi	16	1 (6.25%)	0 (0%)	1 (6.25%)
Eyelid tumors	304	2 (0.66%)	4 (1.32%)	6 (1.97%)
Others	343	0 (0%)	0 (0%)	0 (0%)

We further set the S/CO value at 0.5 levels to see whether similar results were also observed. The seroprevalence in ocular surface diseases, no-ocular surface diseases, and no-ocular diseases was 3.48% (61/1755), 1.95% (245/12550), and 1.98% (116/5852), respectively. The seroprevalence of antibodies to SARS-CoV-2 was also particularly high in the population with ocular surface diseases, similar results were also observed in male and female subgroups (**Table S1**).

**Figure 3:**
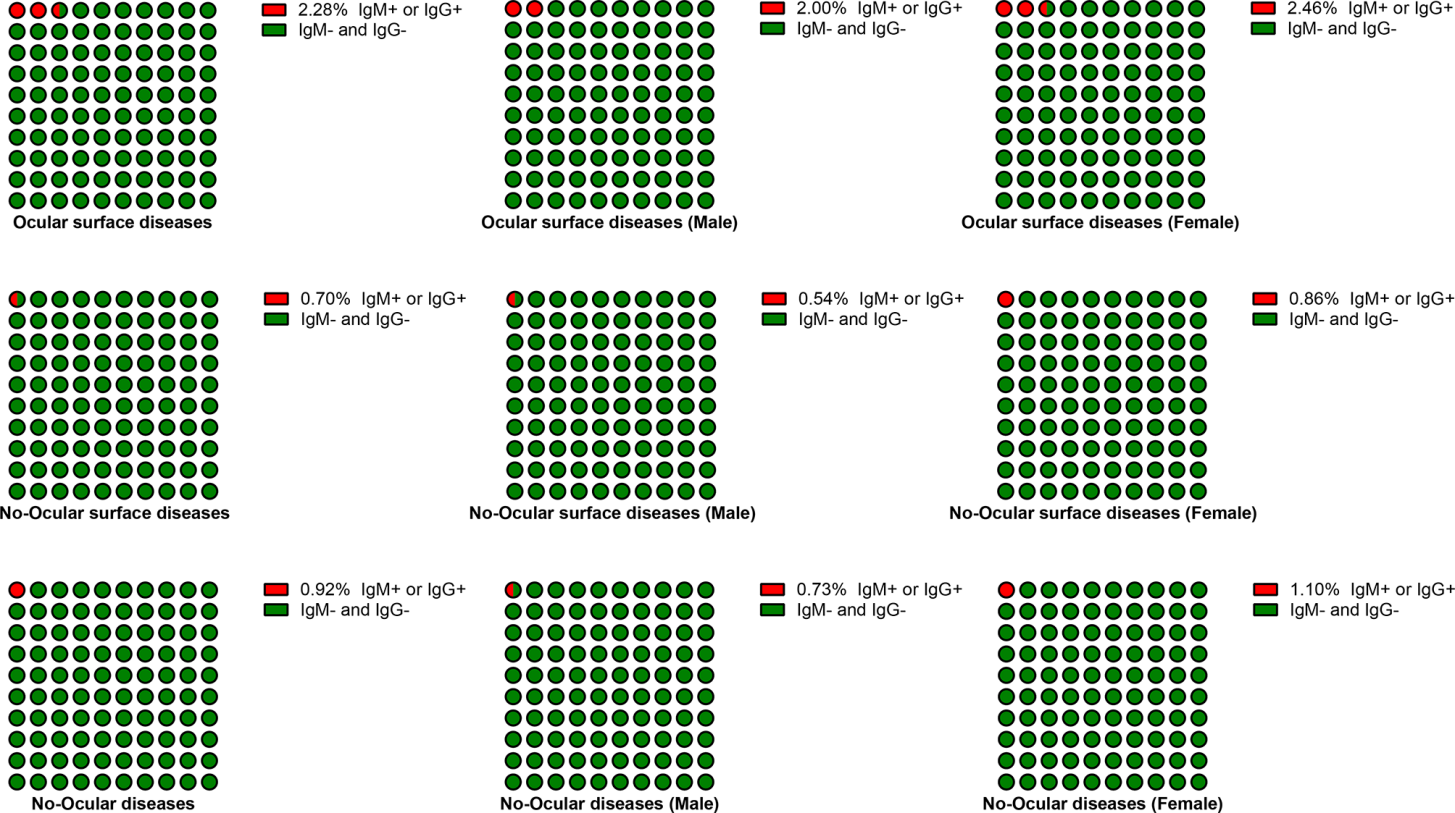
The seroprevalence of antibodies to SARS-CoV-2 in male and female subgroup. Based on their sex, the subjects were categorized into male and female subgroup. The seroprevalence of antibodies to SARS-CoV-2 was particularly higher in ocular surface diseases compared with no-ocular surface diseases and no-ocular diseases in male and female subgroup.

Based on sex, the subjects were categorized into male and female subgroup. The seroprevalence of SARS-CoV-2 was also particularly higher in ocular surface diseases compared with no-ocular surface diseases and no-ocular diseases in male (2.00%, 0.54%, 073%) and female (2.46%, 0.86%, 1.10%) subgroup (p both<0.05) (**Table 2** and **Figure 3**). Similar results were also observed when SARS-CoV-2 IgM ^+^ and IgG ^+^ prevalence was compared among ocular surface diseases, no-ocular surface diseases and no-ocular diseases groups with respect to sex, respectively (p both<0.05) (**Table 2**).

**Figure 4:**
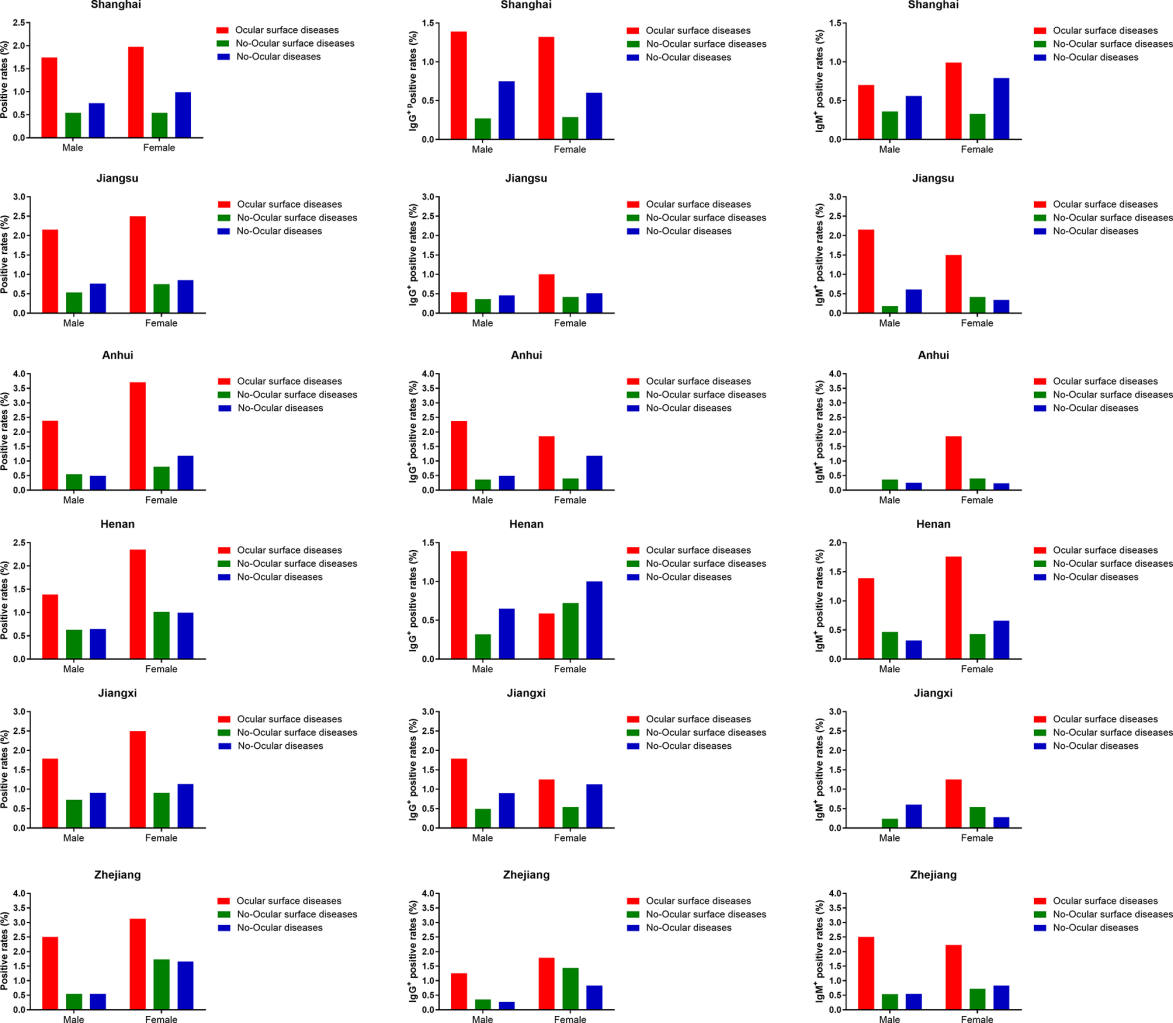
The seroprevalence of antibodies to SARS-CoV-2 in districts subgroup. In each district, the seroprevalence of antibodies to SARS-CoV-2 was particularly higher in ocular surface diseases compared with no-ocular surface diseases and no-ocular diseases in male and female subgroup.

Furthermore, in each district, SARS-CoV-2 prevalence was higher in ocular surface diseases compared with others (p<0.05) (**Table 2**). A similar result was observed when SARS-CoV-2 prevalence was compared among ocular surface diseases, no-ocular surface diseases and no-ocular diseases groups with respect to sex (**Table 2** and **Figure 4**). Similar results were also observed when SARS-CoV-2 IgM ^+^ and IgG ^+^ prevalence was compared among ocular surface diseases, no-ocular surface diseases and no-ocular diseases groups with respect to district and sex, respectively (**Figure 4**).

**Figure 5:**
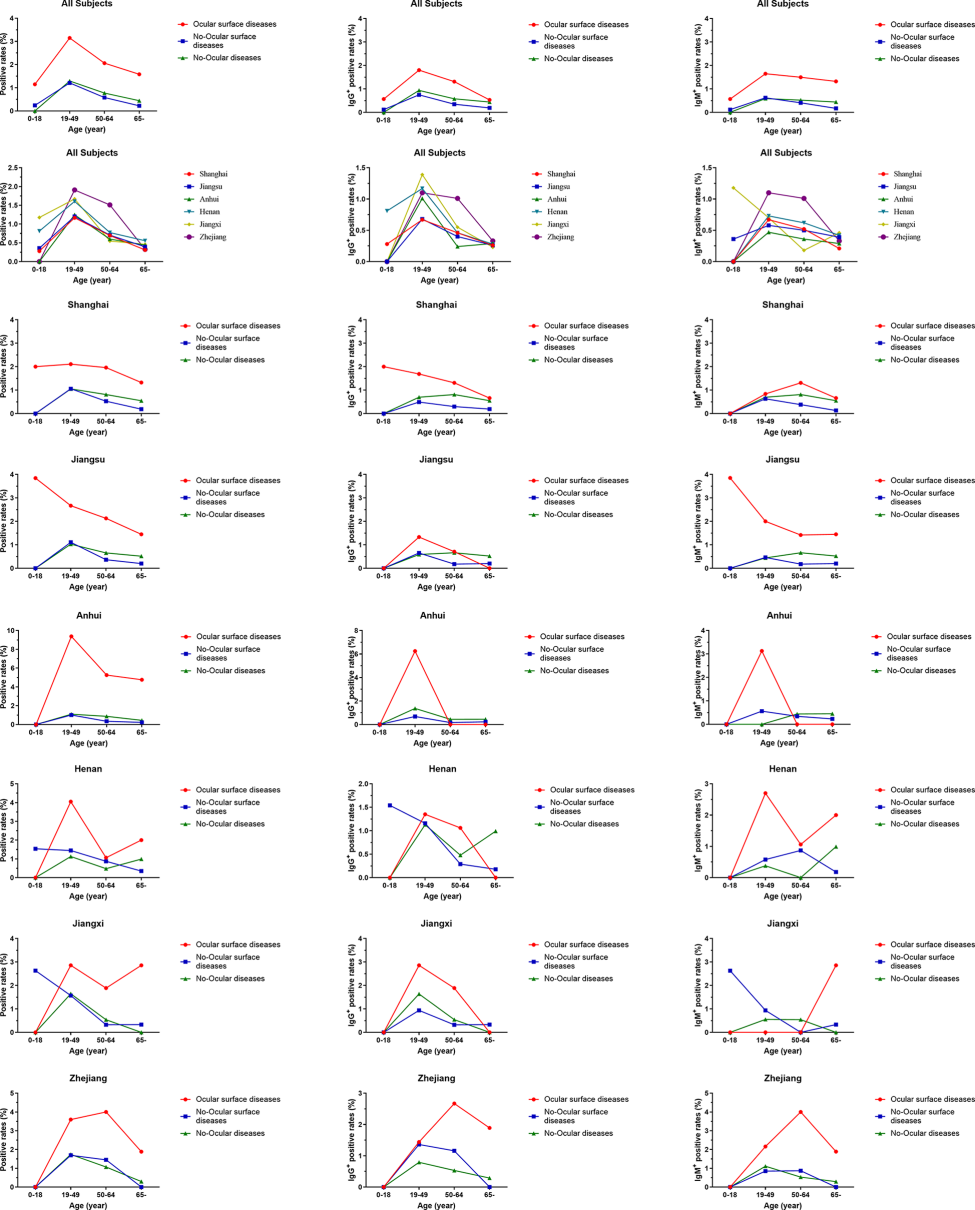
The seroprevalence of antibodies to SARS-CoV-2 in age subgroup. Based on age, all the subjects were further categorized into four subgroups: 0–18 years, 19–49 years, 50–64 years, and >65 years. The seroprevalence of antibodies to SARS-CoV-2 was higher in ocular surface disease with respect to age. A similar result was also observed when SARS-CoV-2 prevalence was compared among ocular surface diseases, no-ocular surface diseases and no-ocular diseases groups in each district.

Based on their age, all the subjects were further categorized into four subgroups: 0–18 years, 19–49 years, 50–64 years, and >65 years. The seroprevalence of antibodies to SARS-CoV-2 was higher in ocular surface disease with respect to age (**Figure 5**). A similar result was also observed when SARS-CoV-2 prevalence was compared among ocular surface diseases, no-ocular surface diseases and no-ocular diseases groups in each district (**Figure 5**). Similar results were also observed when SARS-CoV-2 IgM ^+^ and IgG ^+^ prevalence was compared among ocular surface diseases, no-ocular surface diseases and no-ocular diseases groups with respect to age and district, respectively (**Figure 5**).

**Figure 6:**
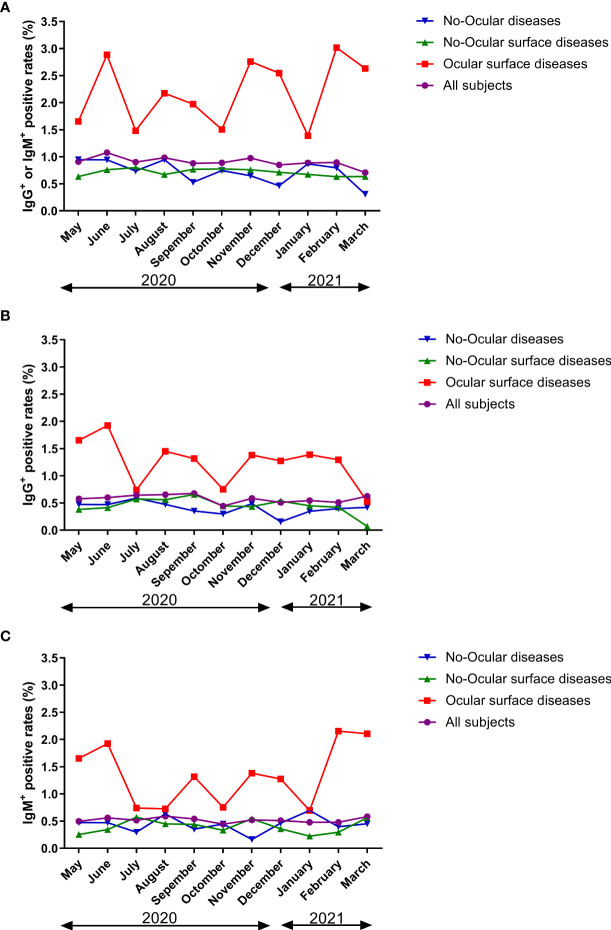
The seroprevalence of antibodies to SARS-CoV-2 in time period subgroup (**A**: IgG^+^ or IgM^+^, **B**: IgG^+^, **C**: IgM^+^). Survey seroprevalence was based on the following numbers of positive tests/sample sizes: 11/1211(May 2020), 27/2506 (June 2020), 14/1553 (July 2020), 15/1528 (August 2020), 13/1481 (September 2020), 14/1574 (October 2020), 15/1537 (November 2020), 15/1767 (December 2020), 13/1466 (January 2021), 28/3134 (February 2021), and 17/2400 (March 2021).

Survey seroprevalence was based on the following numbers of positive tests/sample sizes: 11/1211(May 2020), 27/2506 (June 2020), 14/1553 (July 2020), 15/1528 (August 2020), 13/1481 (September 2020), 14/1574 (October 2020), 15/1537 (November 2020), 15/1767 (December 2020), 13/1466 (January 2021), 28/3134 (February 2021), and 17/2400 (March 2021). In ocular surface disease population, greater than 1.4% of specimens had detectable SARS-CoV-2 antibodies at different times. However, in no-ocular surface disease and no-ocular disease population, less than 0.9% of specimens had detectable SARS-CoV-2 antibodies at different times (**Figure 6A**). Furthermore, similar results were also observed when SARS-CoV-2 IgG ^+^ (**Figure 6B**) and IgM ^+^ (**Figure 6C**) prevalence was compared among three groups at different times.

Logistic regression analyses were performed to identify the association between type of diseases and risk of susceptible to SARS-CoV-2 infection (**Table 4**). Logistic regression analyses revealed that ocular surface diseases [ocular surface diseases *vs.* no-ocular diseases (p=0.001, OR=1.467, 95% CI=1.174-1.834); ocular surface diseases *vs.* no-ocular surface diseases (p<0.001, OR=2.170, 95% CI=1.434-3.284)] were associated with increased risk of susceptible to SARS-CoV-2 infection, no matter IgG^+^ (**Table 5**) but also IgM^+^ (**Table 6**).

**Table 4:** Associations between ocular surface diseases and risk of SARS-CoV-2 infection (IgG^+^ or IgM^+^).

	β	P value	OR	95%CI
Model A				
Age	-0.005	0.485	0.995	0.983 to 1.008
Gender	0.360	0.125	1.434	0.905 to 2.273
Ocular surface diseases	0.383	0.001	1.467	1.174 to 1.834
Model B				
Age	-0.005	0.258	0.995	0.986 to 1.004
Gender	0.298	0.131	1.347	0.915 to 1.982
Ocular surface diseases	0.775	<0.001	2.170	1.434 to 3.284

Model A: Ocular surface diseases vs. No-ocular diseases; Model B: Ocular surface diseases vs. No-ocular surfaces diseases.

**Table 5:** Associations between ocular surface diseases and risk of SARS-CoV-2 infection (IgG^+^).

	β	P value	OR	95%CI
Model A				
Age	-0.013	0.073	0.987	0.973 to 1.001
Gender	-0.042	0.869	0.959	0.583 to 1.577
Ocular surface diseases	0.333	0.011	1.395	1.081 to 1.800
Model B				
Age	-0.007	0.158	0.993	0.983 to 1.003
Gender	-0.213	0.346	0.808	0.518 to 1.260
Ocular surface diseases	0.821	<0.001	2.272	1.399 to 3.688

Model A: Ocular surface diseases vs. No-ocular diseases; Model B: Ocular surface diseases vs. No-ocular surfaces diseases.

**Table 6:** Associations between ocular surface diseases and risk of SARS-CoV-2 infection ((IgM^+^).

	β	P value	OR	95%CI
Model A				
Age	-0.010	0.178	0.990	0.975-1.005
Gender	0.039	0.886	1.039	0.614-1.760
Ocular surface diseases	0.520	<0.001	1.682	1.297-2.182
Model B				
Age	-0.007	0.163	0.993	0.982-1.003
Gender	-0.026	0.909	0.974	0.621-1.528
Ocular surface diseases	1.015	<0.001	2.760	1.720-4.428

Model A: Ocular surface diseases vs. No-ocular diseases; Model B: Ocular surface diseases vs. No-ocular surfaces diseases.

## Discussion

Our results showed that the total positivity rate of IgM and IgG antibodies against SARS-CoV-2 (0.90%) was close to the seropositivity rate (0.8-1.68%) of community settings in several cities of China, as reported in other serological studies (11, 12). Although SARS-CoV-2 seroprevalence studies have previously been published (13–15), this is, to our knowledge, the first study to estimate the seroprevalence of antibodies against SARS-CoV-2 in patients with ocular diseases.

Remarkably, we found that the seroprevalence of antibodies to SARS-CoV-2 was particularly high in the patient population with ocular surface diseases. Clinical and scientific evidence suggests that the human ocular surface is susceptible to SARS-CoV-2 infection (16, 17). A clinician wearing an N95 mask without eye protection was likely infected with SARS-CoV-2 through the eyes (16). An animal study showed that the virus can infect rhesus macaques *via* the conjunctival route (17). Zhou et al. (18) found that 8 of 121 COVID-19 patients studied had ocular symptoms. Wu et al. (2) reported that 12 of 38 COVID-19 patients had ocular manifestations consistent with conjunctivitis, including conjunctival hyperaemia, chemosis, epiphora, and increased secretions. Furthermore, Michael S Deiner et al. (19) also suggest a relationship between the COVID-19 pandemic and internet search patterns for some ocular surface conditions. In a word, it is obviously that there was a significantly association between COVID-19 and ocular surface conditions. However, it is still unknown ocular surface conditions were manifestation of COVID-19, or ocular surface conditions would be a risk factor for getting COVID-19?

Recent studies have suggested that ACE2 and TMPRSS2 are expressed in human conjunctival and corneal cells (5, 20, 21). ACE2 and TMPRSS2 is a major cellular-entry receptor for the SARS-CoV-2 virus, and higher ACE2 expression increases susceptibility to SARS-CoV-2. Collectively, clinical features of reported COVID-19 patients combined with our results indicate that COVID-19 is likely to be transmitted through the ocular surface. Thus, we speculate that ocular surface conditions would be a risk factor for getting COVID-19. Further prospective multi-center longitudinal studies are warranted to elucidate the underlying causal relationship. Furthermore, our previous study suggested that the SARS-CoV-2 receptor ACE2 is expressed in humans, especially in diseased conjunctival tissue (21). Therefore, in patients with ocular diseases—especially ocular surface diseases, such as keratitis and conjunctival cyst—ACE2 is possibly upregulated, making them more susceptible to SARS-CoV-2 infection (21). This may explain why seroprevalence was particularly high in patients with ocular surface diseases in this study.

Our study has certain limitations. First, the specimens were collected for clinical purposes from people seeking health care, potentially causing bias in the results. Second, owing to this study was a large-sample observational study, it is hard for us to obtain detailed demographic data such as diabetes, hypertension, and smoking which might result in bias, and it is hard for us to clarify the causal relationship between ocular surface diseases and SARS-CoV-2 infection. Therefore, multicentre prospective cohort studies are expected to clarify the causal relationship between ocular surface diseases and SARS-CoV-2 infection. Last, the serological assays may have produced false negatives if samples were collected before infected individuals had a serological response.

In conclusion, the seroprevalence of SARS-CoV-2 was particularly high in population with ocular surface diseases, which suggested that there was a significant association between ocular surface disease and SARS-CoV-2 infection. Therefore, increasing awareness of eye protection during the pandemic is necessary, especially for individuals with ocular surface diseases.

## Data Availability Statement

The raw data supporting the conclusions of this article will be made available by the authors, without undue reservation.

## Ethics Statement

The studies involving human participants were reviewed and approved by Eye and ENT Hospital of Fudan University. The patients/participants provided their written informed consent to participate in this study. Written informed consent was obtained from the individual(s) for the publication of any potentially identifiable images or data included in this article.

## Author Contributions

SL, XZ and WC designed this study. SL, YQ, LT, ZW, WC, XZ, and XS prepared and analysed the data. SL, WC, and XZ drafted the manuscript. All co-authors revised the manuscript together. All authors contributed to the article and approved the submitted version.

## Funding

This work was supported by the Shanghai Municipal Commission of Health and Family Planning (20174Y0169 and 201840050), the State Key Program of National Natural Science Foundation of China (81430007), the subject of major projects of National Natural Science Foundation of China (81790641), the Shanghai Committee of Science and Technology of China (17410712500), and the top priority of clinical medicine center of Shanghai (2017ZZ01020) and Shanghai Science and Technology Committee Foundation grant (19411964600), National Natural Science Foundation of China (Grant No. 81770955), Joint research project of new frontier technology in municipal hospitals (SHDC12018103), Project of Shanghai Science and Technology (Grant No.20410710100), Clinical Research Plan of SHDC (SHDC2020CR1043B), Project of Shanghai Xuhui District Science and Technology (2020-015).

## Conflict of Interest

The authors declare that the research was conducted in the absence of any commercial or financial relationships that could be construed as a potential conflict of interest.

## Publisher’s Note

All claims expressed in this article are solely those of the authors and do not necessarily represent those of their affiliated organizations, or those of the publisher, the editors and the reviewers. Any product that may be evaluated in this article, or claim that may be made by its manufacturer, is not guaranteed or endorsed by the publisher.
