# Supplementary material for: Association of Ocular Surface Diseases With SARS-CoV-2 Infection in Six Districts of China: An Observational Cohort Study
Source: Front Immunol. 2021 Aug 6;12:695428. doi: 10.3389/fimmu.2021.695428 (PMC8378230; doi:10.3389/fimmu.2021.695428)
Supplement: Supplementary file 1 [file Table_1.docx]

Table S1. SARS-CoV-2 seroprevalence among ocular surface diseases, No-ocular surface diseases, and No-ocular diseases

|  | n | IgG^+^ | IgM^+^ | IgM^+^ or IgG^+^ |
| --- | --- | --- | --- | --- |
| Ocular surface diseases | 1755 | 40 (2.28%) | 37 (2.11%) | 61 (3.48%) |
| Gender |  |  |  |  |
| Male | 699 | 17 (2.43%) | 15 (2.15%) | 22 (3.15%) |
| Female | 1056 | 23 (2.18%) | 22 (2.08%) | 39 (3.69%) |
| No-ocular surface diseases | 12550 | 151 (1.20%) | 144 (1.15%) | 245 (1.95%) |
| Gender |  |  |  |  |
| Male | 6242 | 66 (1.06%) | 54 (0.87%) | 102 (1.63%) |
| Female | 6308 | 85 (1.35%) | 90 (1.43%) | 154 (2.44%) |
| No-ocular diseases | 5852 | 81 (1.38%) | 62 (1.06%) | 116 (1.98%) |
| Gender |  |  |  |  |
| Male | 2752 | 31 (1.13%) | 29 (1.05%) | 45 (1.64%) |
| Female | 3100 | 50 (1.61%) | 33 (1.06%) | 71 (2.29%) |
| Total | 20157 | 272 (1.35%) | 243 (1.21%) | 422 (2.09%) |

An S/CO value higher than 0.5 for either IgG or IgM was considered positive.
